# Investigating Stimulation Protocols for Language Mapping by Repetitive Navigated Transcranial Magnetic Stimulation

**DOI:** 10.3389/fnbeh.2018.00197

**Published:** 2018-09-10

**Authors:** Nico Sollmann, Sophia Fuss-Ruppenthal, Claus Zimmer, Bernhard Meyer, Sandro M. Krieg

**Affiliations:** ^1^Department of Diagnostic and Interventional Neuroradiology, Klinikum rechts der Isar, Technische Universität München, Munich, Germany; ^2^Department of Neurosurgery, Klinikum rechts der Isar, Technische Universität München, Munich, Germany; ^3^TUM-Neuroimaging Center, Klinikum rechts der Isar, Technische Universität München, Munich, Germany

**Keywords:** cortical mapping, language, navigated transcranial magnetic stimulation, object naming, protocol optimization, stimulation parameters

## Abstract

Navigated transcranial magnetic stimulation (nTMS) is increasingly applied to map human language functions. However, studies on protocol comparisons are mostly lacking. In this study, 20 healthy volunteers (25.7 ± 3.8 years, 12 females) underwent left-hemispheric language mapping by nTMS, combined with an object-naming task, over a cortical spot with reproducible naming errors within the triangular or opercular part of the inferior frontal gyrus (trIFG, opIFG: anterior stimulation) and the angular gyrus or posterior part of the superior temporal gyrus (anG, pSTG: posterior stimulation), respectively. Various stimulation intensities [80, 100, and 120% of the resting motor threshold (rMT)], frequencies (2, 5, 10, and 20 Hz), and coil orientations (in steps of 45°) were evaluated, and the adjustments leading to the highest error rates (ERs), combined with low occurrences of errors due to muscle stimulation, were considered optimal. Regarding anterior stimulation, 100% rMT, 5 Hz, and a coil orientation of 90° or 270° in relation to the respective stimulated gyrus resulted in optimal results. For posterior stimulation, 100% rMT, 10 Hz, and coil orientations of 90° or 270° were considered optimal. Errors due to facial muscle stimulation only played a considerable role during analyses of high-intensity (120% rMT) or high-frequency stimulation (20 Hz). In conclusion, this is one of the first studies to systematically investigate different stimulation protocols for nTMS language mapping, including detailed analyses of the distribution of ERs in relation to various coil orientations considered during neuronavigated stimulation. Mapping with 100% rMT, combined with 5 Hz (anterior stimulation) or 10 Hz (posterior stimulation) and a coil orientation perpendicular to the respective stimulated gyrus can be recommended as optimal adjustments.

## Introduction

Transcranial magnetic stimulation (TMS), a non-invasive method that creates a magnetic field inducing a transient electric field capable of modulating neurons in their activity, is available for neuroscientific and clinical use in human subjects since the middle eighties (Barker et al., 1985; Hallett, 2000). Among its multifarious applications, researchers and clinicians use this technique to induce short-lived language or speech disturbances, depending on the site of stimulation and applied stimulation parameters (Pascual-Leone et al., 1991; Epstein, 1998; Devlin and Watkins, 2007). When performed systematically over several areas of the human brain from outside, it is commonly referred to as language mapping.

While first studies on language mapping by TMS were mostly conducted without simultaneous neuronavigation, the combination of TMS with precise electric-field neuronavigation systems further expanded the role of this evolving technique since we are now able to confirm the cortical area to which TMS is applied very accurately (Ruohonen and Ilmoniemi, 1999; Ruohonen and Karhu, 2010). Thus, navigated TMS (nTMS) became a useful tool both for neuroscience and clinical applications because it allows for tracking of the stimulating coil and spatial identification of stimulated cortical regions, which become so-called language-positive sites when an error is elicited during stimulation under task performance (Lioumis et al., 2012; Picht et al., 2013; Tarapore et al., 2013; Hernandez-Pavon et al., 2014; Rosler et al., 2014). Particularly neurosurgery made advantage of nTMS language mapping in recent years, with neurosurgeons considering such language-positive sites during preoperative planning and intraoperative resection guidance in patients suffering from brain lesions in language-eloquent areas, with the aim of reducing surgery-related functional deficits while maximizing the extent of tumor resection (Picht et al., 2013; Sollmann et al., 2015a, 2018).

However, language mapping by nTMS in patients with brain lesions has repeatedly shown to have a comparably high sensitivity and low specificity when compared to intraoperative direct electrical stimulation (DES) in most studies, which is regarded non-optimal (Picht et al., 2013; Krieg et al., 2014; Ille et al., 2015, 2016; Sollmann et al., 2016b). To improve the specificity of nTMS language mapping, recent studies added nTMS-based diffusion tensor imaging fiber tracking (DTI FT) to cortical mapping, changed the picture-to-trigger interval (PTI) of task presentation, or evaluated different error rate (ER) thresholds for errors induced by stimulation (Krieg et al., 2014; Ille et al., 2015, 2016; Sollmann et al., 2016b). Nevertheless, the overall specificity remained largely unsatisfactory for most stimulated regions (Krieg et al., 2014; Ille et al., 2015, 2016; Sollmann et al., 2016b). Additionally, there have been efforts to improve the underlying stimulation protocol partially responsible for the distribution of induced errors, but such approaches have been limited to specific single adjustments (e. g., the stimulation frequency), only enrolled low subject numbers, or were even performed without neuronavigation before the introduction of nTMS (Pascual-Leone et al., 1991; Epstein et al., 1996; Epstein, 1998; Devlin and Watkins, 2007; Hauck et al., 2015; Sollmann et al., 2015b). Thus, evidence for the dependency of nTMS-induced errors, with respect to different categories of errors and their incidences, on stimulation protocols remains poor. Therefore, the present study aims to investigate various nTMS language mapping protocols by systematically evaluating the effect of stimulation intensity, frequency, and coil orientation on object-naming performance in a group of healthy volunteers.

## Materials and Methods

### Participants and Procedures

The present study was performed in healthy volunteers. Inclusion criteria were German as mother tongue, age of at least 18 years, and right-handedness according to the Edinburgh Handedness Inventory (EHI) (Oldfield, 1971). Subjects with contraindications for magnetic resonance imaging (MRI) at 3 Tesla or language mapping by nTMS (e.g., implanted medical devices such as cardiac pacemaker or deep brain stimulation electrodes), subjects diagnosed with neurological or psychiatric diseases, or pregnant subjects were excluded.

**FIGURE 1:**
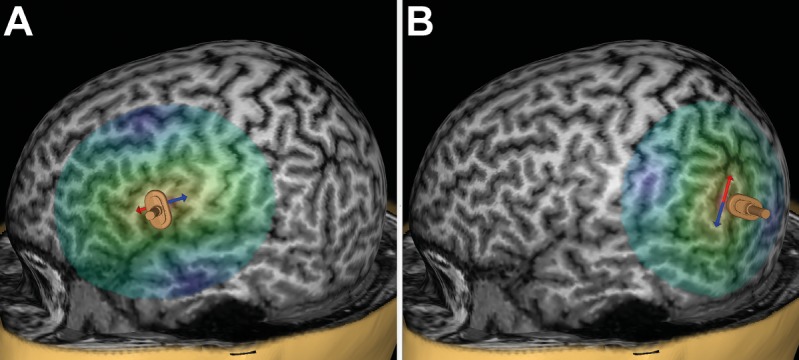
Anterior and posterior stimulation spots. This figure shows a representative example for left-hemispheric anterior stimulation **(A)** and for posterior stimulation **(B)**. For anterior stimulation, the triangular or opercular part of the inferior frontal gyrus (trIFG, opIFG) were considered, whereas posterior stimulation was carried out within the angular gyrus or posterior part of the superior temporal gyrus (anG, pSTG). Regions for anterior and posterior stimulation were visually identified in each subject on the reconstructed three-dimensional (3-D) head model in the stimulation software. Mapping was then performed in steps of 5 to 10 mm to identify a cortical spot leading to reproducible and clear naming errors in each region, and these spots were then used during later stimulation to evaluate the effect of stimulation intensity, frequency, and coil orientation. The arrows indicate the orientation of the induced electric field, which is perpendicular to the respective stimulated gyrus/closest sulcus for anterior stimulation **(A)** and parallel with regards to the respective stimulated gyrus/closest sulcus for posterior stimulation **(B)**. A focal figure-8-shaped stimulation coil with an upward handle position was used, which is shown as a miniature model superimposed on the 3-D head model.

Prior to language mapping, anatomical MRI was performed in all subjects. On a separate day, each volunteer underwent repetitive nTMS (rTMS) to the triangular or opercular part of the inferior frontal gyrus (trIFG, opIFG: anterior stimulation) and the angular gyrus or posterior part of the superior temporal gyrus (anG, pSTG: posterior stimulation) of the left hemisphere (**Figures 1A,B**). The mappings were conducted with different stimulation intensities [80, 100, and 120% of the individual resting motor threshold (rMT)], stimulation frequencies (2, 5, 10, and 20 Hz), and coil orientations (in steps of 45°) during performance of an object-naming task. The different adjustments were applied in randomized order, and nTMS-induced errors were counted and categorized during *post hoc* analyses.

### Anatomical MRI

Anatomical cranial MRI (without intravenous contrast administration) was performed with a 3-Tesla magnetic resonance scanner (Achieva 3T, Philips Medical Systems, The Netherlands B.V.), and the obtained sequence was used to be able to apply neuronavigation during language mapping. The acquired three-dimensional (3-D), T1-weighted gradient echo sequence had the following scanning parameters: repetition time/echo time: 9/4 ms, 1 mm^3^ isovoxel covering the whole head, acquisition time: ∼7 min.

### Language Mapping by nTMS

#### nTMS Setup

Subsequent to the upload of the 3-D T1-weighted gradient echo sequence, the respective subject underwent language mapping by rTMS using a Nexstim eXimia NBS system, version 4.3, with a NexSpeech module (Nexstim Plc., Helsinki, Finland). We used a focal, figure-8-shaped stimulation coil with an upward handle position and automatic overheating protection. The used coil applies biphasic pulses (pulse length: 230 μs) and induces an electric field with a maximum field strength of 172 V/m ± 2% (as measured 25 mm below the coil in a spherical conductor model representing the human head). Maximum stimulator output can be approximately 2.5 × rMT of a hand muscle of a healthy adult by default. The stimulation system enables the visualization of all stimulation areas and simultaneous tracking of the coil due to the combination of the stimulation coil with a neuronavigation unit (Polaris Spectra, Waterloo, ON, Canada) (Ruohonen and Ilmoniemi, 1999; Ruohonen and Karhu, 2010; Lioumis et al., 2012; Picht et al., 2013; Tarapore et al., 2013; Hernandez-Pavon et al., 2014; Rosler et al., 2014). Pulse application can be tracked and controlled on each individual’s reconstructed 3-D head model based on the MRI data set (Ruohonen and Ilmoniemi, 1999; Ruohonen and Karhu, 2010). Furthermore, each stimulation spot is saved and can be reassessed during *post hoc* analyses.

Prior to language mapping by nTMS, the individual rMT was determined. Pregelled surface electrodes (Neuroline 720, Ambu, Ballerup, Denmark) for electromyography recording were placed over the abductor pollicis brevis and abductor digiti minimi muscles, and single stimulation pulses were applied over the left-hemispheric motor cortex at the area of the anatomical hand knob (Rossini et al., 1994; Yousry et al., 1997; Rossini et al., 2015; Sollmann et al., 2017). After identification of the most excitable spot in that area (motor hotspot) with the electric field being oriented perpendicular to the central sulcus during stimulation, the rMT was automatically determined at that spot by using the system’s built-in threshold-hunting procedure, corresponding to the maximum likelihood algorithm (Rossini et al., 1994, 2015; Awiszus, 2003; Sollmann et al., 2017). Percentages of the individual rMT were then used as stimulation intensities during nTMS.

#### Object-Naming Task and Baseline

We used an object-naming task during baseline assessment and language mapping by nTMS, which was displayed on a screen located approximately 60 cm in front of the volunteers (Lioumis et al., 2012; Picht et al., 2013; Tarapore et al., 2013; Hernandez-Pavon et al., 2014; Rosler et al., 2014). The task consisted of 80 drawn, black and white pictures of common living and non-living items and was similar to a set of objects used in the Snodgrass and Vanderwart pictures (Snodgrass and Vanderwart, 1980). The objects were presented at an inter-picture interval (IPI; interval between the display of two objects) of 3.0 s with a display time (DT; time of object presentation on the screen) of 0.7 s per object.

Two consecutive rounds of baseline assessment (object naming without simultaneous nTMS) were conducted to assess the individual naming skills of each volunteer with respect to the assortment of objects (Lioumis et al., 2012; Hernandez-Pavon et al., 2014). The subjects were instructed to name the displayed objects in German as quickly and precisely as possible. All objects that were misnamed or named with a delay according to the investigator’s evaluation were discarded. The number of correctly named objects was documented after the two rounds of baseline testing. This approach facilitates later *post hoc* analyses of nTMS-induced errors (Lioumis et al., 2012; Hernandez-Pavon et al., 2014). The baseline assessments were audio- and video-recorded (Lioumis et al., 2012; Hernandez-Pavon et al., 2014).

#### Mapping Approach

Subsequent to baseline assessments, language mapping by nTMS was performed with the set of remaining, correctly named objects. The IPI and DT were the same as during baseline assessment; thus, the interval between two consecutive stimulation trains was 3.0 s (equal to the IPI), with one stimulation train being applied per every single object presentation. Furthermore, the PTI (onset time; interval between the display of an object and stimulation pulse onset) was set at 0 s, indicating that stimulation started simultaneously with object presentation (Krieg et al., 2014). Pulse application was automatically triggered by the stimulation system with respect to the screening of objects on the screen. The individual set of objects appeared in randomized order until the investigator manually stopped the presentation sequence.

The left-hemispheric trIFG/opIFG (anterior stimulation) and anG/pSTG (posterior stimulation), which were anatomically identified on the reconstructed 3-D head model (**Figures 1A,B**), were mapped in steps of 5–10 mm to identify a cortical spot for each of both cortical regions where nTMS led to reproducible and clear naming errors during task performance (Sollmann et al., 2015b). This was done with 100% rMT, 5 Hz/5 pulses (duration of one stimulation train with these adjustments: 2.0 s), and anterior-posterior (a-p) coil orientation (coil oriented horizontally with respect to a line between the external acoustic meatus and nasion) as the most common nTMS language mapping settings at present (Lioumis et al., 2012; Picht et al., 2013; Tarapore et al., 2013; Hernandez-Pavon et al., 2014; Rosler et al., 2014; Sollmann et al., 2015b). The optimal stimulation intensity, frequency, and coil orientation were then evaluated at these spots with respect to the following approach:

– stimulation intensity: stimulation with 80, 100, and 120% rMT (5 Hz/5 pulses, a-p coil orientation, duration of one stimulation train: 2.0 s), 10 consecutive stimulation trains per intensity at the optimal spot (cortical stimulation spot with high error reproducibility, combined with low occurrences of errors due to muscle stimulations according to the investigator’s evaluation during stimulation) within the trIFG/opIFG and anG/pSTG (3 × 10 × 2 = 60 stimulation trains to test for optimal stimulation intensity).
– stimulation frequency: stimulation with 2 Hz/4 pulses, 5 Hz/10 pulses, 10 Hz/20 pulses, and 20 Hz/40 pulses (optimal intensity, a-p coil orientation, duration of one stimulation train: 2.0 s), 10 consecutive stimulation trains per frequency at the optimal spot within the trIFG/opIFG and anG/pSTG (4 × 10 × 2 = 80 stimulation trains to test for optimal stimulation frequency).
– coil orientation: stimulation with variations in steps of 45° (optimal intensity, optimal frequency, duration of one stimulation train: 2.0 s), 10 consecutive stimulation trains per orientation at the optimal spot within the trIFG/opIFG and anG/pSTG (8 × 10 × 2 = 160 stimulation trains to test for optimal coil orientation).

During mapping, the optimal stimulation intensity and frequency to conduct further stimulation according to the protocol were determined during the ongoing stimulation procedure (Sollmann et al., 2015b). This was based on counting the amount of naming errors elicited during nTMS per setting, and the adjustment leading to the highest amount of naming errors, combined with low occurrences of errors presumably due to muscle stimulations, was defined as optimal for each subject (Sollmann et al., 2015b). Exact analyses determining optimal adjustments for the whole cohort followed the mappings.

The course of mappings applying the different stimulation parameters was randomized for each subject using cards for each adjustment. Prior to language mapping, the first region to map was determined by drawing a card (2 options/cards, 1 draw). Then, the different parameters for testing stimulation intensity (3 options/cards, 2 draws) and frequency (4 options/cards, 3 draws) were individually determined to define the chronological order in which the various settings were tested. Regarding coil orientation, only the initial orientation in relation to a-p coil orientation was drawn (8 options/cards, 1 draw), and the other orientations were tested clockwise (in steps of 45°) with respect to the initially used orientation. Like for the baseline assessments, the whole procedure was recorded (Lioumis et al., 2012; Hernandez-Pavon et al., 2014). After the mapping procedure, each subject was instructed to rate overall perceived discomfort during nTMS according to a visual analog scale (VAS) (Langley and Sheppeard, 1985).

### Video Analyses

*Post hoc* video analyses, which were performed by the investigator who had already conducted the mappings (SFR; certified speech therapist working in a neurological rehabilitation unit), were based on video and audio recordings acquired during the individual baseline assessments and mapping procedures. These data were streamed and each naming performance was compared with the individual baseline to detect disruption of object naming (Lioumis et al., 2012; Picht et al., 2013; Tarapore et al., 2013; Hernandez-Pavon et al., 2014; Rosler et al., 2014). Similar to previous approaches, the induced errors were categorized as follows (Corina et al., 2010; Lioumis et al., 2012; Krieg et al., 2016):

– no-response error: no language production during stimulation.
– performance error: incorrect pronunciation of the target word or slurring/stuttering.
– phonological paraphasias: substitutions, additions, or omissions of phonemes of the target word.
– semantic paraphasias: substitution of the target word by a semantically related word.
– neologism: creation of a non-existent word instead of the target word.

Hesitations (delays in naming the target word during stimulation) were not considered during analyses because we did not use objective measurements of voice reaction times. Furthermore, errors that presumably occurred due to stimulation-induced facial muscle activity according to video analysis or reporting of the volunteers were not included in one of these categories, but were registered separately. For further analyses, we considered all naming errors without hesitations or muscle stimulations (no-response errors, performance errors, phonological paraphasias, semantic paraphasias, and neologisms together), no responses, performance errors, others (phonological paraphasias, semantic paraphasias, and neologisms together), and errors due to muscle stimulation.

### Statistical Analyses

For all statistical analyses, we used GraphPad Prism (version 7.0, GraphPad Software Inc., La Jolla, CA, United States). A *p*-value of <0.05 indicated statistical significance for all statistical tests.

Errors of the different categories were counted to form absolute and relative frequencies for the different adjustments applied during anterior and posterior stimulation, respectively. Descriptive statistics including mean, standard deviation (SD), median, minimum and maximum values were calculated for subject- and mapping-related characteristics and naming errors of the different categories. Discomfort perceived according to the VAS and the electric field strength during nTMS were compared between anterior and posterior stimulation spots using Wilcoxon matched-pairs signed rank tests.

Furthermore, we calculated ERs by dividing the number of errors induced by nTMS by the number of stimulations, which was done for anterior and posterior stimulation as well as for the different adjustments and error categories, respectively (Ille et al., 2015, 2016; Krieg et al., 2016). We then evaluated which adjustments led to the highest ERs per error category for the stimulation intensity (80% rMT, 100% rMT, or 120% rMT), frequency (2, 5, 10, or 20 Hz), and coil orientation (0°/360°, 45°, 90°, 135°, 180°, 225°, 270°, or 315°). With respect to the coil orientation, we evaluated both the angulations in relation to a-p coil orientation and the angulations with respect to the stimulated gyrus/closest sulcus. The adjustments leading to the highest ERs, thus reflecting high reproducibilities of errors, were defined as optimal (except for muscle stimulation where the opposite was true). The incidences of single adjustments when defined as optimal were counted separately for the testing of stimulation intensity, frequency, and coil orientation with respect to the different error categories (e. g., a subject showed an ER of 15.0% for no responses regarding stimulation with 80% rMT, 20.0% for 100% rMT, and 30.0% for 120% rMT – depending on the ER, 120% rMT was defined as the optimal intensity in this subject and the incidence of 120% rMT as an optimal setting increased by 1 in terms of stimulation intensity for the whole cohort). The total number was translated into a relative frequency, given as a percentage and reflecting the fraction of a certain adjustment that led to the highest ER. Chi-squared tests were applied to assess differences between the fractions of optimal adjustments vs. the fractions of non-optimal adjustments, again separately for the stimulation intensity, frequency, and coil orientation for anterior and posterior stimulation and the different error categories.

For the optimal adjustments, we calculated coefficients of variation (CVs) as measures for dispersion regarding error numbers of all errors without hesitations or muscle stimulations. This was again achieved separately for anterior and posterior stimulation and for all subjects together as well as for female and male subjects, respectively. Additionally, were performed Mann–Whitney tests to compare error numbers of this error category between genders.

## Results

### Study Cohort

Twenty healthy volunteers (25.7 ± 3.8 years, 12 females and 8 males) took part in this study. Overall, the stimulation procedure was tolerated well by all volunteers and no adverse events were observed. Stimulation within the trIFG/opIFG was significantly more discomfortable when compared to nTMS to the anG/pSTG (*p* = 0.0001). There was no statistically significant difference in electric field strengths when comparing anterior and posterior stimulation delivered with optimal adjustments (*p* = 0.4980). **Table 1** displays cohort and mapping characteristics.

**Table 1:** Cohort characteristics.

		Mean ± SD	Range
Age (in years)		25.7 ± 3.8	19 – 32
Handedness (according to EHI score)		86.5 ± 10.4	68.4 – 100
No. of correctly named baseline objects (out of 80 objects in total)		70.7 ± 3.7	64 – 75
rMT (% maximum stimulator output)		33.3 ± 6.4	25 – 52
Peeling depth (in mm, for stimulation with optimal parameters)		20.1 ± 0.6	18.2 – 21.5
Discomfort (according to VAS score)	Anterior stimulation	4.8 ± 2.0	1 – 9
	Posterior stimulation	2.9 ± 1.8	0 – 6
Electric field strength (in V/m, for stimulation with optimal parameters)	Anterior stimulation	82.4 ± 19.1	57 – 130
	Posterior stimulation	84.9 ± 19.4	60 – 144

*This table provides an overview of subject- and mapping-related characteristics, including age, handedness according to the Edinburgh Handedness Inventory (EHI), number of correctly named baseline pictures, resting motor threshold (rMT), peeling depth of the anatomical three-dimensional (3-D) magnetic resonance imaging (MRI) sequence used during stimulation, discomfort during mapping according to the visual analog scale (VAS), and electric field strength. Data are provided as mean ± standard deviation (SD) and ranges*.

### Anterior Stimulation

We were able to identify a cortical spot with reproducible naming errors during nTMS in all subjects within the trIFG/opIFG at the beginning of language mapping (**Figure 1A**). Overall, 3,000 nTMS trains were applied to 20 subjects, leading to 295 naming errors of the different categories (9.8%; 14.8 ± 7.7 errors per subject, median: 13.5 errors, range: 5 – 34 naming errors) and 453 errors presumably due to muscle stimulation (15.1%; 22.7 ± 27.7 errors per subject, median: 16.0 errors, range: 0 – 109 errors). Out of the 295 naming errors elicited, 37 were no responses (12.6%), 210 were performance errors (71.2%), 34 were phonological paraphasias (11.5%), 13 were semantic paraphasias (4.4%), and 1 was categorized as neologism (0.3%).

#### Stimulation Intensity

**FIGURE 2:**
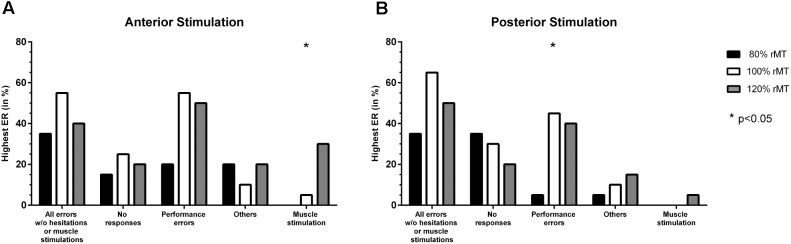
Comparison of error incidences for language mapping with different stimulation intensities. An optimal adjustment for the stimulation intensity (given as % of the individual resting motor threshold [rMT]) was defined as the intensity that resulted in the highest error rate (ER; number of errors/number of stimulations) when compared to the other adjustments (except for errors due to muscle stimulations). The incidences of the adjustments leading to the highest ER were assessed for the whole cohort and different error categories, making it possible that more than one adjustment was counted per person. Panel **(A)** shows how frequent (in %, *y-axis*) a certain stimulation intensity led to the highest ER when compared to the other adjustments for anterior stimulation when considering different error categories (*x-axis*), whereas panel **(B)** shows the results for posterior stimulation. The biggest column represents the optimal adjustment per error category (except for errors due to muscle stimulations where the opposite was true). Statistically significant differences between adjustments are marked by asterisks (^∗^*p* < 0.05).

There was no statistically significant difference between adjustments considering the different error categories except for muscle stimulations (nTMS with 120% rMT led to the highest ER most frequently, *p* = 0.0066; **Figure 2A**). For all errors without hesitations or muscle stimulations, no responses, and performance errors, 100% rMT led to the highest ERs most frequently when compared to stimulation with 80% rMT or 120% rMT (**Figure 2A**).

#### Stimulation Frequency

**FIGURE 3:**
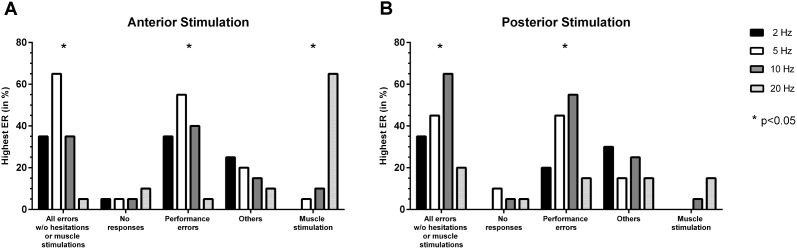
Comparison of error incidences for language mapping with different stimulation frequencies. Similar to the evaluation of optimal stimulation intensities, an optimal adjustment for the stimulation frequency (given in Hz) was determined as the frequency that resulted in the highest error rate (ER; number of errors/number of stimulations) when compared to the other adjustments (except for errors due to muscle stimulations). The incidences of the adjustments leading to the highest ER were assessed for the whole cohort and different error categories, making it possible that more than one adjustment was counted per person. Panel **(A)** shows how frequent (in %, *y-axis*) a certain stimulation frequency led to the highest ER when compared to the other adjustments for anterior stimulation when considering different error categories (*x-axis*), whereas panel **(B)** shows the results for posterior stimulation. The biggest column represents the optimal adjustment per error category (except for errors due to muscle stimulation where the opposite was true). Statistically significant differences between adjustments are indicated by asterisks (^∗^*p* < 0.05).

Stimulation with 5 Hz led to the highest ER for all errors without hesitations or muscle stimulations and performance errors most frequently, with a statistically significant difference in comparison to the other adjustments (*p* = 0.0012, *p* = 0.0081; **Figure 3A**). Highest ERs due to muscle stimulation occurred most frequently in response to stimulation with 20 Hz (*p* < 0.0001; **Figure 3A**).

#### Coil Orientation

**FIGURE 4:**
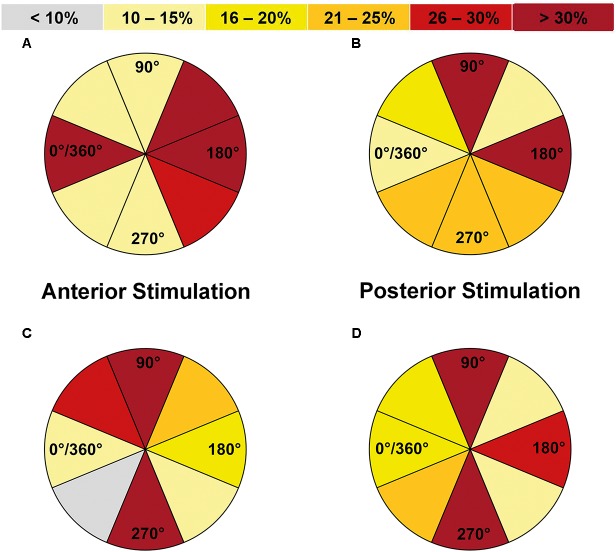
Comparison of error incidences for language mapping with different coil orientations (all errors without hesitations or muscle stimulations). An optimal coil orientation (assessed in steps of 45°) was determined as the orientation that resulted in the highest error rate (ER; number of errors/number of stimulations) when compared to the other adjustments (except for errors due to muscle stimulations). The incidences of the adjustments leading to the highest ER were assessed for the whole cohort and different error categories, making it possible that more than one adjustment was counted per person. Panel **(A)** shows how frequent (in %) a certain coil orientation led to the highest ER in relation to anterior-posterior (a-p) coil orientation when compared to the other adjustments for anterior stimulation when considering all errors without hesitations or muscle stimulations, whereas panel **(B)** shows the results for posterior stimulation. Furthermore, panel **(C)** shows the results in relation to the stimulated gyrus/closest sulcus for anterior stimulation and panel **(D)** shows the analog results for posterior stimulation. *Dark red segments* represent the optimal adjustments under these premises. Corresponding numbers and analyses of statistical significance also for other error categories can be found in **Tables 2**, **3**.

In relation to a-p coil orientation as the most common adjustment in nTMS language mapping, the highest ERs occurred most frequently for stimulation with 0°/360° (equal to a-p coil orientation), 135°, and 180° when considering all errors without hesitations or muscle stimulations (**Table 2** and **Figure 4A**). However, a statistically significant difference between adjustments for different orientations in relation to a-p coil orientation was only observed for performance errors, with 0°/360° in relation to a-p coil orientation being considered optimal (*p* = 0.0313; **Table 2**).

**Table 2:** Distribution of error types for different coil orientations (in relation to anterior-posterior [a-p] coil orientation).

	Coil orientations (in relation to a-p coil orientation)								*p*
	0°/360° %	45° %	90° %	135° %	180° %	225° %	270° %	315° %	
**Anterior stimulation**					
All errors w/o hesitations or muscle stimulations	35	10	15	35	35	30	10	10	0.0992
No responses	0	0	0	5	10	5	0	5	0.4939
Performance errors	45	10	15	30	25	30	10	5	0.0313
Others	10	10	5	25	25	10	0	5	0.1084
Muscle stimulation	10	10	25	5	0	10	5	0	0.1015
**Posterior stimulation**					
All errors w/o hesitations or muscle stimulations	15	20	35	10	35	25	25	25	0.5472
No responses	5	10	0	5	0	10	5	0	0.5602
Performance errors	10	20	40	25	30	25	20	15	0.4591
Others	20	10	5	10	20	10	5	20	0.6009
Muscle stimulation	0	0	0	0	10	0	0	0	0.0481

*An optimal coil orientation for a specific error category was determined as the orientation that resulted in the highest error rate (ER; number of errors/number of stimulations) when compared to the other adjustments (except for errors due to muscle stimulations). The incidences of the adjustments leading to the highest ER are shown in this table (in %) for anterior and posterior stimulation in relation to a-p coil orientation (assessed in steps of 45° in relation to a-p coil orientation, defined as 0°/360°), with the possibility that more than one adjustment was counted per person if two or more adjustments led to the same high ER. *P*-values are derived from testing the number of optimal adjustments counted per orientation against the number of non-optimal adjustments for the different error categories*.

**FIGURE 5:**
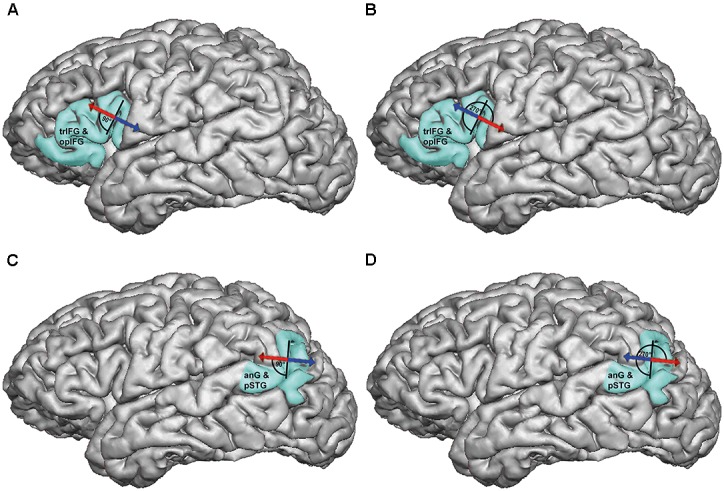
Optimal coil orientations in relation to the stimulated gyrus/closest sulcus (all errors without hesitations or muscle stimulations). This figure shows the optimal coil orientations in relation to the stimulated gyrus/closest sulcus on a standard brain template, which were 90° **(A)** or 270° **(B)** for anterior stimulation within the triangular or opercular part of the inferior frontal gyrus (trIFG, opIFG), and also 90° **(C)** or 270° **(D)** for posterior stimulation within the angular gyrus or posterior part of the superior temporal gyrus (anG, pSTG). The optimal adjustments of coil orientation with respect to the stimulated gyrus/closest sulcus are represented by *dark red segments* in **Figures 4C,D**.

Regarding coil orientations in relation to the respective stimulated gyrus, 90° and 270° led to the highest ERs most frequently for all errors without hesitations or muscle stimulations (**Table 3** and **Figures 4C**, **5A,B**). For this category, the comparison between coil orientations was statistically significant (*p* = 0.0372; **Table 3**).

**Table 3:** Distribution of error types for different coil orientations (in relation to the stimulated gyrus).

	Coil orientations (in relation to the stimulated gyrus)								*p*
	0°/360° %	45° %	90° %	135° %	180° %	225° %	270° %	315° %	
**Anterior stimulation**					
All errors w/o hesitations or muscle stimulations	10	30	40	25	20	10	40	5	0.0372
No responses	0	5	5	10	0	5	0	0	0.4939
Performance errors	10	25	35	20	20	5	40	15	0.1073
Others	5	15	25	10	10	5	5	15	0.4542
Muscle stimulation	25	10	5	0	5	5	5	10	0.1554
**Posterior stimulation**					
All errors w/o hesitations or muscle stimulations	20	20	35	10	30	10	40	25	0.2352
No responses	10	5	5	0	5	5	0	5	0.8420
Performance errors	15	25	35	10	25	15	35	25	0.4591
Others	15	10	20	5	5	10	20	15	0.7121
Muscle stimulation	0	0	0	0	0	0	10	0	0.0481

*An optimal coil orientation for a specific error category was determined as the orientation that resulted in the highest error rate (ER; number of errors/number of stimulations) when compared to the other adjustments (except for errors due to muscle stimulations). The incidences of the adjustments leading to the highest ER are shown in this table (in %) for anterior and posterior stimulation in relation to the respective stimulated gyrus (assessed in steps of 45°), with the possibility that more than one adjustment was counted per person if two or more adjustments led to the same high ER. *P*-values are derived from testing the number of optimal adjustments counted per orientation against the number of non-optimal adjustments for the different error categories*.

For all errors without hesitations or muscle stimulations elicited during nTMS with optimal adjustments, a CV of 55.9% was observed for all subjects together (female subjects: 42.9%, male subjects: 68.5%). However, there was no statistically significant difference in error numbers between genders (*p* = 0.8797).

### Posterior Stimulation

We identified a cortical spot with reproducible naming errors during stimulation within the anG/pSTG in all enrolled subjects (**Figure 1B**). Application of 3,000 nTMS trains in total resulted in 264 naming errors of the different categories (8.8%; 13.2 ± 6.5 errors per subject, median: 11.0 errors, range: 7 – 35 naming errors) and 93 errors presumably due to muscle stimulation (3.1%; 4.7 ± 13.6 errors per subject, median: 0.0 errors, range: 0 – 60 errors). Out of the 264 naming errors elicited, 30 were no responses (11.4%), 182 were performance errors (68.9%), 33 were phonological paraphasias (12.5%), 17 were semantic paraphasias (6.4%), and 2 were classified as neologism (0.8%).

#### Stimulation Intensity

Stimulation with 100% rMT led to the highest ER most frequently for performance errors (*p* = 0.0108; **Figure 2B**). Furthermore, mapping conducted with 100% rMT also resulted in the highest ER most frequently for all errors without hesitations or muscle stimulations, but without a statistical significance when compared to the other intensities (**Figure 2B**). For no responses, 80% rMT led to the highest ERs most frequently, but without statistical significance (**Figure 2B**).

#### Stimulation Frequency

Statistically significant differences were found between adjustments for all errors without hesitations or muscle stimulations and for performance errors, with 10 Hz leading to the highest ER most frequently (*p* = 0.0318, *p* = 0.0185; **Figure 3B**). Concerning no responses, 5 Hz resulted in the highest ER most frequently, but without showing statistical significance (**Figure 3B**).

#### Coil Orientation

Regarding orientations in relation to the a-p coil orientation, the highest ERs occurred most frequently for stimulation with 90° and 180° when considering all errors without hesitations or muscle stimulations (**Table 2** and **Figure 4B**). However, a statistically significant difference between adjustments for different coil orientations with respect to a-p coil orientation was only observed for muscle stimulations (*p* = 0.0481; **Table 2**).

Concerning the coil orientation with respect to the stimulated gyrus, the highest ERs were observed most frequently for stimulation with 90° and 270° when considering all errors without hesitations or muscle stimulations (**Table 3** and **Figures 4D**, **5C,D**). Only for muscle stimulations there was a statistically significant difference between coil orientations (*p* = 0.0481; **Table 3**), but errors due to this were generally very rare in terms of posterior stimulation.

When considering all errors without hesitations or muscle stimulations that were elicited during nTMS with optimal adjustments, a CV of 48.2% was registered for all subjects together (female subjects: 52.7%, male subjects: 39.6%). There was no statistically significant difference in error numbers between genders (*p* = 0.2143).

## Discussion

### Stimulation Intensity

Literature on the distinct impact of stimulation intensities on language or speech performance is rare. Some early procedures were based on successful trials inducing disruptions by using only one specific or a small range of intensities, mostly applying a fixed stimulator output intensity or increasing output intensities instead of percentages of the individually determined rMT for stimulation (Pascual-Leone et al., 1991; Epstein, 1998; Devlin and Watkins, 2007). Since output intensities cannot be directly compared between systems, our results cannot directly be related to these findings. However, studies by Epstein et al. (1996, 1999) used the rMT during stimulation, showing that speech arrests, similar to the no-response errors of our present study, preferentially appear at supra-threshold intensities. In detail, Epstein et al. (1996) started with a stimulation intensity of 120% rMT and increased it, if necessary, up to 150% rMT to induce speech arrests, with the average intensities needed ranging between 113 and 143% rMT for all subjects together. Moreover, in a later study, the authors described the occurrence of speech arrests during stimulation with an average intensity of 116% rMT, with ranges of 100–137% rMT (Epstein et al., 1999). Such findings led to the suggestion that stronger stimulation is probably more capable of producing prominent effects on language function when compared to low-intensity stimulation, with supra-threshold intensities probably showing speech arrests more prominently (Epstein et al., 1996, 1999; Epstein, 1998; Devlin and Watkins, 2007). Correspondingly, a recent study of our group suggested 120% rMT to be more effective than stimulation with 80 or 100% rMT in inducing errors during object naming in a small cohort of five subjects (Sollmann et al., 2015b).

In our present study, optimal results were achieved for nTMS delivered with 100% rMT for both anterior and posterior stimulation (**Figures 2A,B**), which seems to be in contrast to these earlier findings. This was not only true for no responses regarding anterior stimulation (**Figure 2A**), but also for all errors together without hesitations or muscle stimulations or performance errors (**Figures 2A,B**), thus categories not considered in most of the aforementioned earlier studies. The discrepancy between our present and earlier findings might be associated with the use of neuronavigation, which has not yet been available in Epstein’s studies (Epstein et al., 1996, 1999). Optimal coil placement, including perpendicular coil angulation with respect to the skull, might lead to occurrence of errors already at lower intensities thanks to optimized electric field induction that can be achieved with modern electric-field neuronavigation systems (Ruohonen and Ilmoniemi, 1999; Ruohonen and Karhu, 2010; Sollmann et al., 2016a). However, our previous small series among five volunteers using nTMS presented 120% rMT as an optimal setting overall, although 100% rMT also led to frequent errors in most subjects (Sollmann et al., 2015b). The small cohort size and high inter-individual variability may play essential roles regarding this discrepancy. Furthermore, it can be argued that the rMT might not necessarily be the optimal value to use for regulating the intensity during nTMS language mapping. This value is derived from stimulating the motor system, but generally acknowledged as a standardized and widely distributed measure of cortical excitability in general, thus being used as a reference value regarding stimulation intensity for multifarious TMS applications (Rossini et al., 1994; Lefaucheur et al., 2014; Picht, 2014; Rossini et al., 2015). As such, the rMT seems to be a reasonable parameter for adjusting intensities during language mapping, but studies considering other individualized parameters such as the active motor threshold, for example, are lacking. Furthermore, the modern electric-field neuronavigation systems may enable nTMS language mapping driven by the electric field strength individually measured at the cortical surface or at deeper levels, but approaches in this direction have not yet been published according to the authors’ knowledge.

### Stimulation Frequency

In contrast to the stimulation intensity, various frequencies have already been evaluated in earlier investigations. In this context, Pascual-Leone et al. (1991), who performed TMS with frequencies of 8, 16, and 25 Hz, found that 8 and 16 Hz led to speech arrests most sufficiently in their study cohort (Pascual-Leone et al., 1991). Epstein et al. (1996), who used five different frequencies (2, 4, 8, 16, and 32 Hz), demonstrated that slower repetition rates of TMS resulted in clearer language impairment, especially with regards to speech arrests. By using 4–8 Hz, the best ratio of efficacy to pain occurred, and a clearer distinction between speech arrests and dysarthria resulting from stimulation-induced contraction of cranial muscles was observed (Epstein et al., 1996). Correspondingly, they showed that stimulation with 4–8 Hz elicited clear speech arrests in all subjects, whereas stimulation with 16 or 32 Hz did not even lead to such errors in half of the enrolled subjects (Epstein et al., 1996). Furthermore, a recent study using nTMS reported that stimulation with 5 Hz evokes a higher number of errors in total when considering different categories, whereas 7 Hz is able to specifically evoke more speech arrests (Hauck et al., 2015). However, a comparatively high frequency of 20 Hz showed to lead to reproducible errors in our previous small series investigating different adjustments of stimulation, which was not true to the same level for 5, 7, or 10 Hz (Sollmann et al., 2015b).

Overall, 5 Hz (anterior stimulation) and 10 Hz (posterior stimulation) showed optimal results during nTMS in the present study (**Figures 3A,B**). Of note, the category of no responses preferentially occurred at 20 Hz (anterior stimulation) and 5 Hz (posterior stimulation), thus probably supporting the findings of higher frequencies being more capable of leading to speech arrests (**Figures 3A,B**) (Pascual-Leone et al., 1991; Hauck et al., 2015). Discrepancies regarding the efficiency of either low- or high-frequency stimulation in eliciting errors could be related to the various pulse numbers applied at a specific frequency, which have not been the same in previous investigations (Pascual-Leone et al., 1991; Epstein et al., 1996; Hauck et al., 2015; Sollmann et al., 2015b). Furthermore, muscle twitching induced by stimulation is a relevant issue when evaluating different stimulation frequencies, which is particularly true for stimulation to anterior areas since the temporal muscle becomes affected. Thus, especially anterior stimulation with a high frequency of 20 Hz led to a considerable fraction of errors due to muscle stimulations (**Figure 3A**). In this context, muscle stimulation can lead to difficulties during error categorization and discomfort, both potentially hampering proper evaluation of stimulation sessions in terms of language errors, which has been observed also in the studies that preferred higher over lower stimulation frequencies (Pascual-Leone et al., 1991; Sollmann et al., 2015b). However, despite muscle stimulations were observed, they were not specifically categorized and counted, thus possibly leaving the impression that more errors occur during high-frequency stimulation because errors due to muscle stimulation were partially also encountered (Pascual-Leone et al., 1991; Sollmann et al., 2015b). Although errors due to cortical stimulation might not always be easily differentiated from errors due to stimulation-related muscle twitching, the present study emphasizes the need to consider muscle stimulations as a separate category. This should lead to increased awareness during analyses and might reduce confounding of results.

### Coil Orientation

To the authors’ knowledge, this is one of the first studies to systematically evaluate coil orientation in the context of nTMS language mapping. Here we evaluated both coil orientations in relation to the respective stimulated gyrus and in relation to a-p coil orientation as the most common setting in recent studies on nTMS language mapping (Lioumis et al., 2012; Picht et al., 2013; Tarapore et al., 2013; Hernandez-Pavon et al., 2014; Rosler et al., 2014; Sollmann et al., 2015b). Previously, Epstein et al. (1996), who performed TMS with coil orientations of 0° and 90°, concluded that 0° is the more favorable setting. However, further orientations were not evaluated in their study, leaving it questionable whether other adjustments not assessed might have even resulted in optimized results (Epstein et al., 1996). More importantly, neuronavigation has not been available, thus leaving the relation of these two adjustments to the individual gyral architecture unclear (Epstein et al., 1996). While early studies were not able to precisely evaluate various coil orientations on a fine-grained level due to missing neuronavigation, a small series of our group investigated various orientations during nTMS language mapping for the first time in steps of 45° related to a-p orientation and the stimulated gyrus (Sollmann et al., 2015b). As a result, the study came to the conclusion that a-p coil orientation was not the optimal adjustment, thus challenging current nTMS language mapping procedures (Sollmann et al., 2015b).

Against this background, the present study provides a mixed picture when it comes to the evaluation in relation to a-p coil orientation: 0°/360°, 135°, and 180° showed to be optimal for anterior stimulation, whereas 90° and 180° were optimal for posterior stimulation (**Table 2** and **Figures 4A,B**). However, when considering the individual gyral anatomy at the cortical spot stimulated as considered in evaluations related to the respective stimulated gyrus, a clear finding with optimal adjustments of 90° and 270° to this gyrus for both anterior and posterior stimulation was revealed (**Table 3** and **Figures 4C,D**, **5A–D**). This finding seems to correspond to the well-known observations during TMS motor mapping where strongest motor-evoked potentials occur when the stimulating coil is oriented perpendicular to the central sulcus during stimulation of the precentral gyrus (Brasil-Neto et al., 1992; Mills et al., 1992). As suggested based on these observations, an electric field relative to the columnar functional organization of the cortex is crucial for successful cortical responses due to TMS (Fox et al., 2004; Richter et al., 2013). This is even suggested to be more relevant than the strength of the applied electric field and should be considered a primary determinant of cortical excitation (Fox et al., 2004). Although specifically presented as a column-based model of the impact of electric fields on the motor cortex, a similar pattern may take also effect during language mapping and, thus, at preferentially different anatomical spots. Thus, to be effective, the induced electric field has to be aligned with the column, while an electric field oriented perpendicular to the column becomes ineffective (Fox et al., 2004). This may explain why we found optimal results for perpendicular orientation of the coil with respect to the respective stimulated gyrus. As a consequence regarding nTMS language mapping, future studies may rather aim for orientation of the stimulating coil with reference to the respective gyrus than strict a-p coil orientation in order to achieve optimal results. However, we have to acknowledge the fact that this may not always be possible especially for clinical or spatially widespread mappings due to increased efforts, enhanced manual skills, and time restrictions.

### Significance and Limitations

The present investigation and previous studies suggest that small changes in stimulation adjustments can already have a considerable impact on language performance (Epstein et al., 1996, 1999; Epstein, 1998; Devlin and Watkins, 2007; Sollmann et al., 2015b). Hence, studies on protocol optimization are highly relevant since language mapping results are dependent on the stimulation protocol chosen. This study reflects the most systematic evaluation of different stimulation adjustments to date using modern electric-field neuronavigation during stimulation. Furthermore, it is one of the first studies to provide data on the impact of various stimulation adjustments on the incidence of different error categories that are commonly observed during nTMS language mapping combined with an object-naming task. As the study’s main result, we are able to offer a protocol recommendation for nTMS language mapping. For anterior stimulation, 100% rMT, 5 Hz, and a coil orientation of 90° or 270° in relation to the respective stimulated gyrus may be used to elicit high ERs combined with low occurrences of errors due to muscle stimulation (**Table 3** and **Figures 2A**, **3A**, **4C**, **5A,B**). For posterior stimulation, 100% rMT, 10 Hz, and coil orientations of 90° or 270° should be considered to achieve optimal results (**Table 3** and **Figures 2B**, **3B**, **4D**, **5C,D**).

However, at this point, we have to recall that comparisons between single adjustments did not lead to statistically significant results for all error categories, although ER distributions in favor of single adjustments seem evident (**Figures 2–5**). Thus, few subjects may show optimal results for other than the recommended parameters, which underlines the importance of individualization of stimulation parameters in each subject, if possible. In neuroscientific use among healthy subjects or for spatially circumscribed nTMS mappings, this may be achievable and should still be aimed for, with the current recommendations serving as a good starting point for further individual adjustments in single subjects. In clinical use and for spatially widespread nTMS mappings especially among patients suffering from brain tumors, the present recommendations may replace current procedures that commonly apply partially different protocols, especially in terms of coil orientations (Picht et al., 2013; Tarapore et al., 2013; Krieg et al., 2014, 2017). In patients, extensive protocol individualization prior to nTMS language mapping seems too time-consuming, and may not be performed regularly when it comes to clinical applications with time constraints and patients that may not tolerate exhaustive investigations.

Regarding the error types considered during analyses, we closely referred to recent investigations on stimulation mappings among healthy subjects and patients with brain tumors (Corina et al., 2010; Lioumis et al., 2012; Picht et al., 2013; Hernandez-Pavon et al., 2014; Krieg et al., 2016). In this context, hesitations were not incorporated as they can still be regarded as a comparatively untrustworthy error category, with some authors not taking them into consideration (Corina et al., 2010; Lioumis et al., 2012). Yet, it is still not completely clear how to judge such hesitation errors in nTMS language mapping. On the one hand, they can principally be regarded as artifacts due to an oversensitive investigator or might reflect a mild form of a no-response error. On the other hand, they might indeed not only represent an incomplete no-response error, but could constitute a separate type of error. Since the present study aimed to provide an optimized protocol for nTMS language mapping that is based on common procedures that actually mostly consider all errors without hesitations or muscle stimulations, the exclusion of hesitations seems justified. However, the distinct origin and nature of hesitation errors elicited by nTMS should be further explored in upcoming studies. Such studies should apply objective voice-onset measurements during analyses.

We have to acknowledge some limitations of the present study. First, stimulation was only performed at two left-hemispheric cortical areas, whereas most clinically oriented procedures conduct more widespread mappings (Picht et al., 2013; Tarapore et al., 2013; Krieg et al., 2014, 2017). Thus, evaluation of the present adjustments may also be performed in other regions and the other hemisphere in future studies. Such future investigations should also consider a control region or sham stimulation, thus enabling attribution of specific naming errors to stimulation effects more reliably. Second, as the present study focused on stimulation protocol comparisons, we have not yet included mappings among patients with brain tumors and, therefore, altered intracranial anatomy. It has to be determined whether tumor-induced changes in anatomy also require further changes in stimulation parameters, at least when mappings reach tumor borders or tumor infiltration zones with possible derangement of gyral architecture and subcortical fibers. Third, we also did not investigate how stimulation with various adjustments and, thus, different distributions of ERs in comparison to current protocols may influence the sensitivity and specificity of nTMS language mapping, which could be improved by the presented optimal adjustments. This has to be determined during awake procedures using intraoperative DES, as done previously for current protocols using mainly 100 – 120% rMT and 5 –7 Hz to elicit errors (Picht et al., 2013; Tarapore et al., 2013; Krieg et al., 2014, 2017). It is important to validate errors observed during nTMS by the current gold-standard method of functional mapping (Ojemann et al., 1989; Sanai and Berger, 2010). However, because intraoperative DES represents a highly invasive technique not applicable in healthy volunteers, the results of the present study regarding optimal adjustments should be implemented in clinical preoperative nTMS mapping among patients with brain tumors, followed by intraoperative DES to assess correlations.

## Conclusion

This is one of the first studies systematically investigating various stimulation adjustments using modern electric-field neuronavigation with the aim of optimizing current nTMS language mapping protocols. Stimulation with 100% rMT, combined with 5 Hz (anterior stimulation within the trIFG/opIFG) or 10 Hz (posterior stimulation within the anG/pSTG) and a coil orientation perpendicular to the stimulated gyrus, are recommended to achieve optimal results. As the next step, these adjustments should be applied in the preoperative setting among patients with language-eloquent brain tumors, followed by intraoperative DES to evaluate whether improved sensitivity and specificity of nTMS language mapping can be achieved with these settings.

## Availability of Data and Material

All data used for analysis are presented in the manuscript. The discussion and conclusions only rely on the data presented. Raw mapping data can be provided upon request.

## Ethics Statement

The experimental setup was approved by the local ethics committee (Ethikkommission der Fakultät für Medizin der Technischen Universität München, Munich, Germany; registration number: 338/16S) and was conducted in accordance with the Declaration of Helsinki. Written informed consent was obtained from all subjects prior to the mapping procedures.

## Author Contributions

NS designed the experiments, acquired, handled, analyzed (including statistics), and interpreted the data, performed the literature research, drafted the manuscript, and read and approved the final version. SF-R acquired, handled, analyzed, and interpreted the data, performed the literature research, drafted the manuscript, and read and approved the final version. CZ and BM designed the experiments, acquired and handled the data, supervised the study, and read and approved the final version. SK designed the experiments, acquired, handled, and interpreted the data, performed the literature research, drafted the manuscript, supervised the study, and read and approved the final version.

## Conflict of Interest Statement

SK is a consultant for Brainlab AG (Munich, Germany) and Nexstim Plc. (Helsinki, Finland). The remaining authors declare that the research was conducted in the absence of any commercial or financial relationships that could be construed as a potential conflict of interest.

## Abbreviations

3-D: three-dimensional
a-p: anterior-posterior
anG: angular gyrus
CV: coefficient of variation
DES: direct electrical stimulation
DT: display time
DTI FT: diffusion tensor imaging fiber tracking
EHI: Edinburgh Handedness Inventory
ER: error rate
IPI: inter-picture interval
MRI: magnetic resonance imaging
nTMS: navigated transcranial magnetic stimulation
opIFG: opercular part of the inferior frontal gyrus
pSTG: posterior part of the superior temporal gyrus
PTI: picture-to-trigger interval
rMT: resting motor threshold
rTMS: repetitive navigated transcranial magnetic stimulation
SD: standard deviation
TMS: transcranial magnetic stimulation
trIFG: triangular part of the inferior frontal gyrus
VAS: visual analog scale
